# Nogo Receptor 1 (*RTN4R*) as a Candidate Gene for Schizophrenia: Analysis Using Human and Mouse Genetic Approaches

**DOI:** 10.1371/journal.pone.0001234

**Published:** 2007-11-28

**Authors:** Ruby Hsu, Abigail Woodroffe, Wen-Sung Lai, Melloni N. Cook, Jun Mukai, Jonathan P. Dunning, Douglas J. Swanson, J. Louw Roos, Gonçalo R. Abecasis, Maria Karayiorgou, Joseph A. Gogos

**Affiliations:** 1 Department of Neuroscience, Columbia University, New York, New York, United States of America; 2 Department of Epidemiology, Center for Statistical Genetics, University of Michigan, Ann Arbor, Michigan, United States of America; 3 Department of Physiology and Cellular Biophysics, College of Physicians and Surgeons, Columbia University, New York, New York, United States of America; 4 Department of Psychology, The University of Memphis, Memphis, Tennessee, United States of America; 5 Department of Anatomy and Neurobiology, University of Tennessee Health Science Center, Memphis, Tennessee, United States of America; 6 Department of Psychiatry, University of Pretoria and Weskoppies Hospital, Pretoria, Republic of South Africa; 7 Department of Biostatistics, Center for Statistical Genetics, University of Michigan, Ann Arbor, Michigan, United States of America; 8 Department of Psychiatry, College of Physicians and Surgeons, Columbia University, New York, New York, United States of America; James Cook University, Australia

## Abstract

**Background:**

NOGO Receptor 1 (RTN4R) regulates axonal growth, as well as axon regeneration after injury. The gene maps to the 22q11.2 schizophrenia susceptibility locus and is thus a strong functional and positional candidate gene.

**Methodology/Principal Findings:**

We evaluate evidence for genetic association between common *RTN4R* polymorphisms and schizophrenia in a large family sample of Afrikaner origin and screen the exonic sequence of *RTN4R* for rare variants in an independent sample from the U.S. We also employ animal model studies to assay a panel of schizophrenia-related behavioral tasks in an *Rtn4r*-deficient mouse model. We found weak sex-specific evidence for association between common *RTN4R* polymorphisms and schizophrenia in the Afrikaner patients. In the U.S. sample, we identified two novel non-conservative *RTN4R* coding variants in two patients with schizophrenia that were absent in 600 control chromosomes. In our complementary mouse model studies, we identified a haploinsufficient effect of *Rtn4r* on locomotor activity, but normal performance in schizophrenia-related behavioral tasks. We also provide evidence that Rtn4r deficiency can modulate the long-term behavioral effects of transient postnatal N-methyl-D-aspartate (NMDA) receptor hypofunction.

**Conclusions:**

Our results do not support a major role of *RTN4R* in susceptibility to schizophrenia or the cognitive and behavioral deficits observed in individuals with 22q11 microdeletions. However, they suggest that *RTN4R* may modulate the genetic risk or clinical expression of schizophrenia in a subset of patients and identify additional studies that will be necessary to clarify the role of *RTN4R* in psychiatric phenotypes. In addition, our results raise interesting issues about evaluating the significance of rare genetic variants in disease and their role in causation.

## Introduction

Axonal growth and regeneration is restricted during the maturation of the central nervous system (CNS) as well as after injury, and several lines of evidence indicate that myelin-associated proteins play a critical role in these processes. Early seminal studies by David and Aguayo, 1981 [Ref. 1] demonstrated that the CNS environment contains inhibitory factors which limit growth, and a number of myelin-associated growth inhibitory factors have since been identified, including oligodendrocyte-myelin glycoprotein (OMgp), myelin-associated glycoprotein (MAG), Nogo-A (RTN4), and most recently, Ephrin-B3. Notably, despite their distinct molecular structures, OMgp, MAG, and RTN4 all share a common receptor, Nogo Receptor 1 (RTN4R) [2]–[4]. RTN4R, a glycosylphosphatidylinositol-linked (GPI-linked) cell surface molecule, forms a heteromeric receptor complex with either LINGO-1 and p75, or LINGO-1 and TROY (a tumor necrosis factor (TNF) receptor family member). Activation of RTN4R initiates a cascade that leads to the activation of RhoA and, ultimately, the inhibition of axonal growth [5]–[7]. Given that it acts as a convergence point for three separate factors, which inhibit neurite outgrowth and regeneration, RTN4R has generated much interest as a target for therapeutic intervention following CNS injury [2]–[4], [8].

The human *RTN4R* gene is located within the 22q11.2 locus where relatively common hemizygous microdeletions occur at a frequency of 1 in 5000 live births [9]. The majority of these deletions are *de novo* events and occur on different haplotype backgrounds [10]. The physical phenotype of the 22q11.2 deletion is broad and variable and most frequently includes congenital heart defects, velopharyngeal defects, and thymic hypoplasia. In addition, most patients display a pattern of cognitive impairments and behavioral deficits, including deficits in working memory, conflict monitoring, visuospatial short-term memory, and executive visual attention [11]–[14].

In addition, a range of neuroanatomical abnormalities such as reduced total brain volume, enlarged ventricles, and white matter abnormalities [15], [16] has been described in some 22q11.2 microdeletion carriers. Both syndromic and non-syndromic patients with the deletion also show extremely high frequencies of psychiatric illness, especially schizophrenia: children with the deletion are 25–30 times more likely to develop schizophrenia or schizoaffective disorder by early adulthood [17], [18], and 22q11.2 microdeletions account for ∼2% of schizophrenia cases in Caucasian populations [19]. Children with this microdeletion are also reported to have impaired sensorimotor gating [20], which is considered an endophenotype of several psychiatric disorders including schizophrenia, as well as a high incidence of emotional problems including anxiety, depression, social withdrawal, and obsessive-compulsive behaviors [13].

The possibility that RTN4R deficiency contributes to the psychiatric symptoms associated with the 22q11.2 microdeletion, in particular, is intriguing and supported by some preliminary human genetic and gene expression studies. Although, collectively, the human genetic and animal model studies designed to identify schizophrenia susceptibility genes from the 22q11.2 region have implicated primarily three genes: proline dehydrogenase (*PRODH), ZDHHC8* and catechol-*O*-methyltransferase (*COMT*) [21]–[25] and their interactions [26], [27], a study by Liu et al. [22] presented suggestive evidence that common variants located at the 3′ end of the *RTN4R* gene are associated with schizophrenia in patient samples from the U.S. and South Africa. There has been one attempt to replicate these initial findings in the Han Chinese population, with negative results [28]. However, two preliminary reports described sex-specific associations between schizophrenia and common variants in the *RTN4R* gene [29], as well as its ligand *RTN4* [30]. In addition, Sinibaldi et al. [31] reported two rare non-conservative sequence variants in the *RTN4R* gene in an Italian sample of 120 schizophrenia patients that were absent in a sample of 200 controls. Moreover, alterations in the levels of RTN4R or two RTN4R ligands have been described in postmortem analyses of brains from individuals with schizophrenia. Specifically, microarray expression studies and single-gene quantitative RT-PCR studies indicate a down-regulation of MAG in at least some schizophrenia cohorts [32], [33] and one preliminary study suggested that levels of RTN4 mRNA are also increased in the cortex of some individuals with chronic schizophrenia [34]. Finally, a recent meta-analysis of several expression profiling studies revealed a ∼10% decrease (*P* = 0.019) of *RTN4R* transcript levels in brains of individuals with schizophrenia (www.stanleygenomics.org). It is unclear, however, whether the observed changes constitute part of the genetic diathesis in schizophrenia or represent a reactive response. Here, we undertake a comprehensive multi-pronged approach to explore the possibility that *RTN4R* is a schizophrenia susceptibility gene from the 22q11.2 locus.

## Results

### Search for rare *RTN4R* coding variants

**Figure 1:**
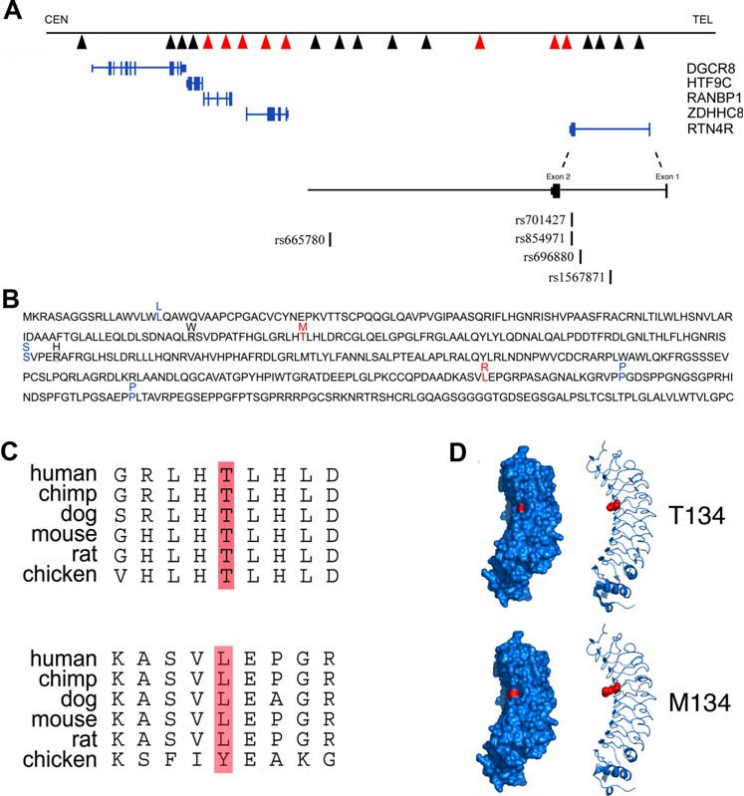
Genetic variation at the *RTN4R* locus. (A) Location of human *RTN4R* within chromosomal region 22q11.2. Exons are represented by blue bars. The rs numbers of the SNPs typed in the present study are shown. SNPs genotyped by Liu et al. [22] are indicated by arrowheads (those SNPs that showed significant association with schizophrenia are shown in red arrowheads). The association observed with the three SNPs at the 3′end of *RTN4R* was independent from the association observed more proximally in the vicinity of the *ZDHHC8* gene [22]. (B) Complete amino acid sequence of *RTN4R*. Rare coding variants identified in the U.S. schizophrenia sample are shown in blue (synonymous) or red (missense). Two missense variants reported by Sinibaldi et al. [31] are shaded in grey. (C) Evolutionary conservation across species of the missense variants identified in this study at amino acid positions 134 (top) and 347 (bottom). (D) Surface (left) and cartoon (right) representation of the crystal structure of the ligand-binding ectodomain of RTN4R. The variant at residue 134, drawn in red, is shown as the wild-type threonine (top) and the mutated methionine (bottom). Prepared using Pymol (DeLano Scientific, LLC, San Francisco, CA).

The RTN4R protein is encoded by two exons: exon I, which codes for the first 7 amino acids, and exon II, which encodes amino acids 8–473 (**Fig. 1A**). We sequenced exon II of *RTN4R* in 208 individuals with schizophrenia from the U.S. (European ancestry) and identified five variants. Of these five variants, three were synonymous changes (L18L, S192S, P394P), and two resulted in non-conservative changes (T134M, L347R) in evolutionary conserved amino acids (**Fig. 1B, C**). Each of these five variants was identified in single individuals except for S192S, which was found in two individuals. We also sequenced exon II of *RTN4R* in a sample of 300 control individuals from the U.S. and found only two synonymous changes (L18L, S192S). The S192S variant was identified in a single individual, whereas the L18L variant was found in two individuals. Previously, Sinibaldi et al. [31] reported two non-conservative sequence variants in the *RTN4R* gene (R119W and R196H) in an Italian sample of 120 schizophrenia patients that were absent in 200 controls. In our sample, we did not find these two variants in our patient sample, but we did find the synonymous L18L variant reported in the same study.

The RTN4R protein is comprised of two domains: an extracellular N-terminal ectodomain containing eight leucine-rich repeats (LRRs) and a membrane-anchored C-terminal domain containing a glycosylphosphatidylinositol (GPI) anchor sequence [35]. The ectodomain is both necessary and sufficient for binding the RTN4 ligand [35] and the C-terminal domain containing the GPI sequence and the connecting “stalk” is required for interaction with at least one of the co-factors, p75 [36]. The T134M variant we identified in one female patient is located in the concave face of the ligand-binding ectodomain of RTN4R, within the fourth LRR. Based on the crystal structure of the soluble ectodomain [37], [38] the T134 residue is found on the concave face within a negatively charged patch of amino acids including D111, D114, C138, D139, which forms a slight local depression which could be important for ligand or partner interactions [38] (**Fig. 1D**). The other non-synonymous variant we identified in one male patient, L347R, is positioned within the “stalk” of RTN4R that is required for p75 interaction.

### Association analysis

Given the difficulties in assessing the significance of association to rare variants, primarily due to a lack of power [39], we sought to obtain additional supportive data by testing for independent association with common variants of *RTN4R* (**Fig. 1A**) in families with schizophrenia. Previously, we provided weak evidence for an association between *RTN4R* variation and susceptibility to schizophrenia by identifying three distinct variants located at the 3′ end of the *RTN4R* gene that were preferentially transmitted in individuals with schizophrenia in two independent, family-based samples from the U.S. (N = 106 triads) and South Africa (N = 93 triads) [22]. Here, we expand the South African sample to 312 families by including an additional 219 families with at least one schizophrenic subject (see **Table S1** for study population characteristics). Since it has been suggested that variants in *RTN4R* may affect schizophrenia susceptibility differently in males and females we conducted both combined and sex-stratified analyses.

To facilitate comparison, three of the five single nucleotide polymorphisms (SNPs) tested in the present study (rs665780, rs701427, and rs696880) were also included in the earlier study [22]. Two of these SNPs previously showed weak evidence for association in that study: rs665780 in the Afrikaner families only (*P* = 0.04) and rs701427 in the U.S. families only (*P* = 0.02). Four of the five SNPs in this study are within the *RTN4R* intron and one is located 3′ of the gene. All SNPs are common with a minor allele frequency of at least 20% (see **Table 1** for SNP positions and minor allele frequencies). The average (±SD) R^2^ and D' between the ten pairs of SNPs are 0.33 (±0.42) and 0.62 (±0.48), respectively. In the HapMap subjects of European descent (CEU), these five SNPs tag the common (minor allele frequency >10%) SNPs in *RTN4R* with an average R2 (±SD) of 0.75 (±0.39). Three of the five SNPs (rs701427, rs854971, and rs696880) are separated by less than 2-kb and are in high linkage disequilibrium (LD) with each other (R^2^≥0.90), (see **Table 2** for pair-wise values). This group of SNPs, however, shows relatively low LD with the two flanking SNPs (rs665780 and rs1567871).

**Table 1:** Position and minor allele frequency of SNPs

SNP	Position (in Mb)	Position (in Gene)	Major/Minor Alleles	Minor Allele Frequency
rs665780	18.560	3′	T/C	.21
rs701427	18.608	intronic	C/A	.34
rs854971	18.608	intronic	G/A	.34
rs696880	18.610	intronic	A/G	.36
rs1567871	18.616	intronic	C/T	.24

**Table 2:** Pair-wise LD values

	rs665780	rs701427	rs854971	rs696880	rs1567871
rs665780	-	<.01	<.01	<.01	<.01
rs701427	.05	-	**.99**	**.90**	.16
rs854971	.05	**1.00**	-	**.91**	.16
rs696880	.06	**.99**	**1.00**	-	.17
rs1567871	.08	**.99**	**.99**	**.98**	-

Transmission disequilibrium test (TDT) analysis did not find evidence for unequal allelic transmission ratios at any of the tested variants in the combined sample (data not shown). Application of the more powerful linkage and association modeling in pedigrees (LAMP) analysis (**Table 3**) on the combined sample also provided no evidence for unequal allelic transmission. In a sex-stratified analysis, SNP rs696880 showed the most significant association in females (Sch1: RR_A_ = 0.74, *P* = .064; Sch2: RR_A_ = 0.73, *P* = .046) and non-significant trends were also obtained with the other two linked SNPs, rs701427 and rs854971. In affected males, suggestive evidence for association was observed at rs701427 (Sch1: RR_C_ = 1.19, *P* = .10; Sch2: RR_C_ = 1.21, *P* = .019), as well as at the two SNPs (rs696880 and rs854971) in high LD with it. Interestingly, the alleles of rs701427, rs854971, and rs696880 that appear to increase susceptibility to schizophrenia in males show a trend towards decreasing the risk in females, consistent with the observed lack of association in the combined sample. A Bonferroni correction for multiple SNPs and affection statuses results in a *P*-value threshold of 0.0042. Therefore, after correcting for multiple testing, none of these results are significant.

**Table 3:** LAMP results for individual SNPs

	SNP	Allele	Sch1		Sch2	
			RR	*P*-Val	RR	*P*-Val
All	rs665780	T	1.12	-	1.10	-
	rs701427	C	0.97	-	0.98	-
	rs854971	G	0.97	-	0.98	-
	rs696880	A	0.96	-	0.96	-
	rs1567871	C	0.98	-	1.01	-
Females	rs665780	T	1.23	-	1.23	-
	rs701427	C	0.73	.062	0.74	.054
	rs854971	G	0.74	.070	0.74	.064
	rs696880	A	0.74	.064	0.73	.046
	rs1567871	C	1.12	-	1.18	-
Males	rs665780	T	1.02	-	1.00	-
	rs701427	C	1.19	-	1.21	.019
	rs854971	G	1.18	-	1.20	.021
	rs696880	A	1.17	-	1.18	.029
	rs1567871	C	0.91	-	0.91	-

* RR = risk ratio; *P*-Val = *P*-value of T_LD_ statistic.

* Bonferroni corrected *P*-value cutoff = .0050.

When we estimate the haplotype frequencies in our population using an Expectation Maximization (E-M) algorithm, we observe six common haplotypes (frequency ≥5%) and one additional haplotype with a frequency ≥1%. The TDT analysis reveals a proportionate transmission of all haplotypes (data not shown). Similarly, the LAMP analysis does not detect any association between a haplotype and schizophrenia either in the combined or in the sex-stratified sample (see **Table 4** for the results from the LAMP analyses).

**Table 4:** LAMP results for haplotype analysis (frequency>.05)

	Haplotype* [Table-fn nt104] [Table-fn nt105] [Table-fn nt106]	Freq	Sch1		Sch2	
			RR	*P*-Val	RR	*P*-Val
All	TCGAC	.34	0.94	-	0.94	-
	TAAGC	.27	1.05	-	1.01	-
	TCGAT	.17	1.10	-	1.10	-
	CAAGC	.07	0.97	-	1.06	-
	CCGAC	.07	0.95	-	0.98	-
	CCGAT	.06	0.89	-	0.82	-
Females	TCGAC	.34	0.93	-	0.92	-
	TAAGC	.27	1.28	.053	1.29	.055
	TCGAT	.17	0.89	-	0.87	-
	CAAGC	.07	1.30	-	1.27	-
	CCGAC	.07	0.46	.054	0.59	-
	CCGAT	.06	0.86	-	0.77	-
Males	TCGAC	.34	0.96	-	0.98	-
	TAAGC	.27	0.90	-	0.84	-
	TCGAT	.17	1.20	.058	1.23	.061
	CAAGC	.07	0.78	-	0.90	-
	CCGAC	.07	1.32	.083	1.28	.081
	CCGAT	.06	0.94	-	0.91	-

* Alleles for rs665780, rs701427, rs854971, rs696880, and rs1567871, respectively.

* Rare haplotypes (not included in analysis: TCGGC (.018); CCGGC (.007); TCGGT (.001); TAGAC (.001); TAAGT (<.001).

* RR = risk ratio; *P*-Val = *P*-value of T_LD_ statistic.

* At α-level of .05, Bonferroni corrected *P*-value is .0042.

### Generation of *Rtn4r*-deficient mice

In order to complement our human genetic studies (see Discussion), we generated a mouse model that is deficient in the *Rtn4r* gene and probed for deficits in phenotypic components, which include endophenotypes associated with schizophrenia that can be modeled reliably in mice [40].

**Figure 2:**
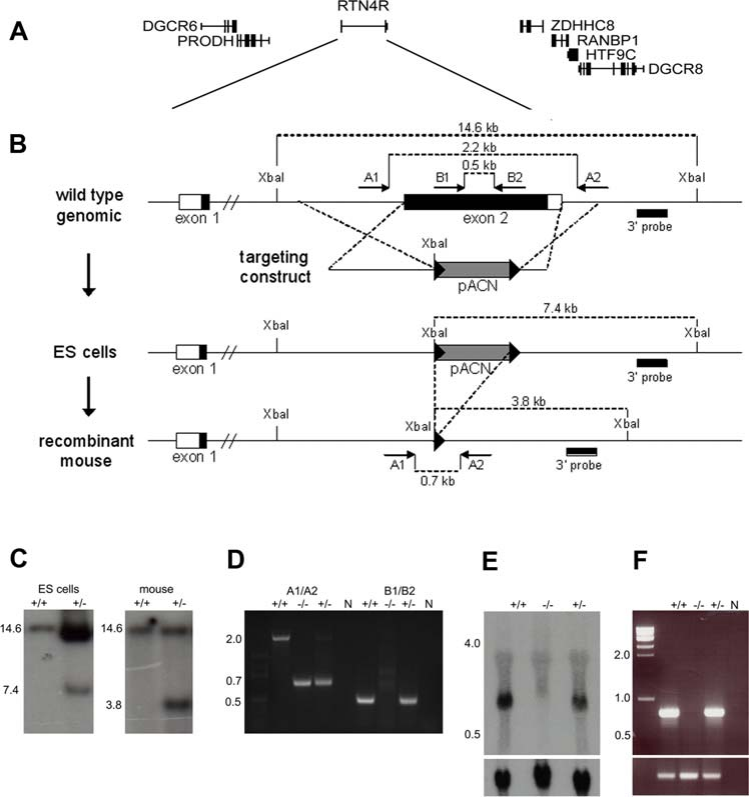
Targeting the *Rtn4r* locus for homologous recombination. (A) Gene map of the *Rtn4r* locus at mouse chromosome 16. (B) Exon II of *Rtn4r* was replaced by the self-excisable pACN cassette including the *neo* gene for selection. Genomic DNA was digested with *Xba*I and the 3′ probe was used for southern blot analysis. A PCR strategy was designed to genotype offspring using primer sets A1/A2 and B1/B2. (C) Genomic southern blot of ES cell DNA and tail biopsies. Wild-type fragments were 14.6-kb, recombinant fragments from ES cells were 7.4-kb, and recombinant fragments from tail biopsies after pACN cassette excision were 3.8-kb. (D) PCR genotyping of tail biopsies using primer sets A1/A2 and B1/B2. Wild-type mice yielded 2.2-kb and 0.5-kb fragments from sets A1/A2 and B1/B2 respectively; homozygotes yielded 0.7-kb and no fragment, respectively; and heterozygotes yielded 0.7-kb and 0.5-kb fragments, respectively. (E) Northern blot of total brain RNA using a *Rtn4r* 3′UTR probe. (F) RT-PCR of total brain RNA using isoform-specific primers for *Rtn4r* exon II.

The mouse ortholog of *RTN4R* is located in the syntenic region of the human 22q11.2 locus that lies on mouse chromosome 16. All human genes except for one are represented in this region, although the order of the genes is different [41]. As a result of this rearrangement, the *Rtn4r* mouse gene is located between the *Prodh* gene (∼40-kb proximal) and the *Zdhhc8* gene (∼70-kb distal) (**Fig. 2A**), both of which have been implicated by human genetic studies as strong candidate susceptibility genes for schizophrenia [21]–[23]. To minimize the possibility that genetic modification of the *Rtn4r* locus could affect expression of the two neighboring genes, we designed and implemented a gene targeting strategy (**Fig. 2B–F**) to replace exon II of *Rtn4r* with the self-excisable selection cassette that includes the *neo* gene selectable marker (pACN) [40]. Excision of the *neo* gene, following germline transmission, ensures that any observed phenotype is due to the deletion rather than any long-range transcriptional effects of the selection cassette [42]. Homozygous *Rtn4r* mutant mice were viable and fertile and have normal brain morphology by gross morphological inspection, as well as finer morphometric calculations of cell densities, laminal thickness and the thickness of the corpus callosum (see **Text S1** and **Fig. S1**).

### Behavioral characterization of *Rtn4r*-deficient mice at baseline

We characterized wild-type, heterozygous, and homozygous *Rtn4r* mutant mice at baseline using tasks designed to assess domains known to be perturbed in schizophrenia, including anxiety, sensorimotor gating, learning and memory, and behavioral despair. *Rtn4r*-deficient mice demonstrated normal neurological signs and normal pain threshold levels using the hot plate assay (data not shown). In addition to locomotor activity and anxiety-related tasks, we assessed a) sensorimotor gating using the prepulse inhibition (PPI) assay; b) cognitive performance using contextual and cued fear conditioning, as well as a working memory task; and c) a depression-relevant behavior using the tail-suspension assay. For a description of the equipment and procedures used, see **Text S2**.

#### Open field and anxiety-relevant tasks

**Figure 3:**
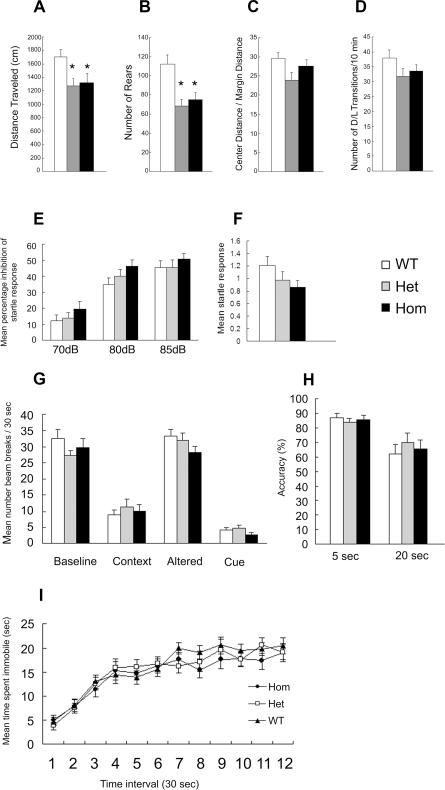
Behavioral characterization of *Rtn4r*-deficient mice. (A) Total distance traveled, (B) mean number of rears, and (C) ratio of center traveled distance/margin traveled distance in a 20-min open field test. (D) Number of dark-to-light transitions in a 10-min dark/light transition test. (E) PPI using combinations of one startle level (120 dB) and three prepulse levels (70, 80 and 85 dB). (F) Startle response at 120 dB. (G) Contextual and cued fear-conditioning paradigm. Activity (beam breaks) was measured at baseline, after reintroduction into the same context where training occurred (context), at baseline in an altered context (altered), and after presentation of the cued conditioned stimulus in the altered environment (cue). (H) Accuracy in a working memory test after a 5-sec or 20-sec inter-trial delay using a delayed alternation T-maze assay. (I) Mean time spent immobile in a tail suspension test. All results shown are from combined sexes. All data are represented as mean±S.E.M.

In the open field assay there was a significant effect of genotype on the total distance traveled in the open field [F(2, 60) = 4.192, *P*<0.02] and number of rears (vertical movements) [F(2, 60) = 9.14, *P*<0.001] (**Fig. 3A, B**). Interestingly, reductions in distance traveled and rears were observed in both the homozygous mutant and heterozygous mice in comparison to wild-type littermates suggesting that the gene could be haploinsufficient with respect to these behaviors. There were no differences in habituation of locomotor activity in the open field (not shown). Differences in the percentage of distance traveled in the central area of the open field, an index of anxiety-relevant behavior, fell short of significance [F(2, 60) = 2.11, *P* = 0.071] (**Fig. 3C**). In the light/dark test, which also assays for anxiety-relevant behaviors, we found no genotype or genotype X sex effects on the number of entries into each compartment (**Fig. 3D**) or on the percentage of time animals spent in the light compartment of the light/dark apparatus (data not shown).

#### Sensorimotor Gating

Sensorimotor gating is typically evaluated using the PPI paradigm, a common measure of pre-attentive processing [40]. Abnormal PPI has been described in individuals with schizophrenia, as well as in children with the 22q11.2 microdeletion syndrome [20]. In this model, the response to a startle-eliciting stimulus, such as a loud tone, is attenuated if the stimulus is preceded within a few hundred milliseconds by a lower-intensity stimulus, or pre-pulse [43], [44]. We evaluated startle response to auditory stimuli as well as inhibition of the startle response elicited by pre-pulses. There were no genotype or genotype X sex differences in inhibition of the startle response at any of the prepulse levels of 70, 80 and 85 dB (**Fig. 3E**). In addition, in control experiments, there were no genotype or genotype X sex effects on acoustic startle response (120 dB) (**Fig. 3F**). Notably, although there was no significant effect of genotype, homozygous mutants tended to show slightly more inhibition of startle response than wild-type littermate control mice, as well as slightly less startle response.

#### Associative learning and memory

We used the Pavlovian conditioned fear paradigm to assess associative learning and memory in *Rtn4r*-deficient mice (**Fig. 3G**). We evaluated both contextual conditioning, as measured by suppressed activity in the context where training occurred, and cued conditioning as measured by suppression of activity in a new environment in which the conditioned stimulus (CS) is presented.

##### Baseline activity

There were no genotype differences or genotype X sex effects in baseline activity during the training phase of fear conditioning. There were also no differences in activity levels after the third presentation of the unconditioned stimulus (US) and tone pairing.

##### Contextual Conditioning

There were no genotype or genotype X sex effects in activity during contextual conditioning.

##### Altered Context

There were no genotypic differences in activity levels in the altered context test, which is both amygdala- and hippocampus-dependent [F(2, 60) = 2.11, *P* = 0.13]; however, there was a significant genotype X sex interaction in activity levels in the altered environment [F(2, 60) = 4.2, *P*<0.02].

##### Cued Conditioning

In the cued version of the test, which requires the amygdala but not the hippocampus [45], there were no genotype or genotype X sex effects in the suppression of activity in response to the conditioned stimulus.

#### Working memory

We used the T-maze delayed alternation task [46], [47] to examine whether deficiency in Rtn4r-mediated signaling activity is associated with changes in spatial working memory. Working memory is defined as the ability to maintain and manipulate information transiently in the service of other cognitive processes to guide behavior [48]. Working memory is frequently impaired in patients with schizophrenia (often prior to or at the onset of the illness), as well as in a portion of their non-schizophrenic, first-degree relatives [49], [50]. Impaired working memory has also been noted in individuals with the 22q11.2 microdeletion syndrome [12]–[14]. *Rtn4r*-deficient mice learned the 5-sec delay T-maze task during 6 consecutive training days and performed as well as wild-type littermate control mice. In addition, there were no genotypic differences or genotype X sex interactions in their T-maze based working memory performance at both 5-sec and 20-sec delays (**Fig. 3H**).

#### Behavioral despair

High rates of depressive symptoms have been observed in patients with schizophrenia [51] and children with the 22q11.2 microdeletion [12]. We assayed for behavioral despair, an index of depression-like behavior in rodents, using a tail suspension test [52]. The test is based on the fact that animals subjected to the short-term, inescapable stress of being suspended by their tail will develop an immobile posture. Various antidepressant medications reverse the immobility and promote the occurrence of escape-related behavior [53]. No genotypic differences or genotype X sex interactions in total time spent immobile on the tail suspension task were observed (**Fig. 3I**). A repeated measures ANOVA also showed that there were no genotypic differences or genotype X sex interactions in time spent immobile over intervals of the test session.

### Behavioral characterization of *Rtn4r*-deficient mice following transient postnatal NMDAR blockade

Recent work using an independent animal model suggests that RTN4R may function in the mammalian CNS to stabilize neural circuits in early postnatal development [54]. It is therefore conceivable that RTN4R deficiency facilitates the effect of other schizophrenia susceptibility genes or disease-related pathological processes by destabilizing relevant neural circuits during development. Such a contribution might not be detectable with the study design outlined above but may require additional genetic or pharmacological challenges. Aberrant NMDA receptor-mediated glutamatergic transmission has been implicated in psychiatric disorders such as schizophrenia, and several pharmacological modeling approaches have been established based on this hypothesis [40]. In one of them, transient inhibition of NMDAR signaling during early postnatal development produces long-lasting behavioral deficits including deficits in domains such as cognitive flexibility, working memory and sensorimotor gating [55], [56]. We employed this model to ask whether Rtn4r deficiency modulates the long-term behavioral effects of postnatal NMDA receptor hypofunction. In our experimental paradigm, mouse pups were given injections of the NMDA channel blocker MK-801 during two distinct periods: postnatal days (P) 7–10, which corresponds to a critical period of sensitivity for the NMDAR system, and P11–14, which follows the critical period [57], [58]. Sensorimotor gating, as well as spontaneous alternation (an index of normal working memory) and motoric activity in the Y-maze were then tested in adulthood.

#### Spontaneous alternation

**Figure 4:**
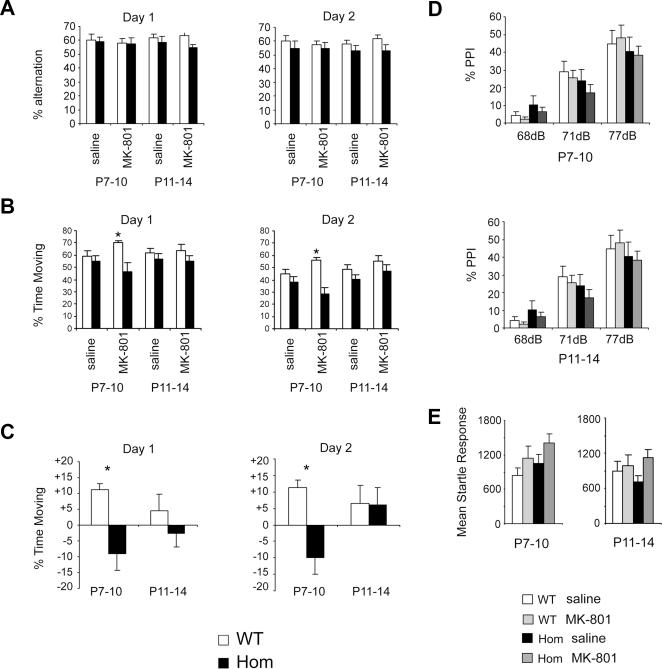
Behavioral characterization of adult *Rtn4r*-deficient mice following transient postnatal NMDAR blockade. (A, B) Percentage of alternation (A) and % time moving (B) observed in an 8-min session of a Y-maze spontaneous alternation assay, repeated over 2 days. Animals were injected with MK-801 or saline for 4 consecutive days during either P7–10 or P11–14; behavioral testing was conducted at P62 and P63. (C) Relative effect of MK-801 injection normalized to saline injection on time spent moving. (D) Average percentage of PPI (measured at P91) in animals injected during either P7–10 or P11–14. (E) Mean startle response to a 40 ms, 120 dB acoustic stimulus in animals injected during either P7–10 or P11–14. Startle response was measured at P91.

Adult homozygous *Rtn4r*-deficient mice and their wild-type littermates showed, as expected, a tendency to spontaneously alternate but there were no significant differences in spontaneous alternation in the Y-maze due to genotype or drug treatment (**Fig. 4A**). However, we observed a significant genotype X treatment interaction (*P*<0.05) in the percentage of time moving, a measure of locomotor activity. Specifically, a significant genotype effect was observed in animals that were injected with MK-801 but not saline at P7–10 (*P*<0.05). Interestingly, this effect was limited to animals treated at P7–10. Animals that underwent the same treatment at P11–14 showed no significant genotype X treatment interaction (**Fig. 4B**). Further analysis showed that this difference emerged because while neonatal MK-801 treatment at P7–P10 induced an increase in locomotor activity in the wild-type mice in adulthood, it resulted in a decrease in locomotor activity of adult *Rtn4r*-deficient mice (**Fig. 4C**).

#### Acoustic startle and prepulse inhibition

There were no overall significant differences in prepulse inhibition or acoustic startle response due to genotype or drug treatment (**Fig. 4D, E**). Previously, Harris et al. [**56**] found that neonatal MK-801 administration resulted in a greater decrease in PPI levels in adult females only. However, we found no significant sex effect in both acoustic startle response and prepulse inhibition.

## Discussion

Several lines of evidence suggest that oligodendroglial function and myelin maintenance are disturbed in schizophrenia [reviewed in Davis et al. [59]]. These include imaging and neurocytochemical evidence, changes in white matter, myelin-related gene abnormalities, and morphologic abnormalities in the oligodendroglia observed in brains of individuals with schizophrenia. The relation of these findings to the pathogenesis of the disease is still uncertain. Most intriguing is the possibility of a relation between deficits in myelination and function of the prefrontal cortex, a region that modulates many functions impaired in schizophrenia and whose myelination is completed during late adolescence and early adulthood, a period when overt symptomatology of schizophrenia most commonly emerges. In this context, the location of *RTN4R* within the replicated and highly penetrant 22q11.2 schizophrenia susceptibility locus makes the gene a prime candidate for influencing disease susceptibility.

We tested common SNPs and haplotypes in and around *RTN4R* in a large sample of Afrikaner families. Given that Afrikaners are a relatively genetically and environmentally homogenous population, we expected higher power in our analysis, making disease susceptibility alleles easier to identify. Our analyses did not identify SNPs or haplotypes that were segregating with the disease in the overall sample. Stratification by sex provided weak evidence for an association between *RTN4R* and schizophrenia, which did not survive a conservative Bonferroni correction.

We also identified rare non-synonymous changes within the *RTN4R* gene of schizophrenia patients but not in unaffected controls. This observation appears to lend some support for a role of *RTN4R* in disease susceptibility. However, the major limitation in conducting association analyses of rare variants is that it is difficult to obtain enough statistical power to unambiguously interpret their results. In our study, the sample of several hundred chromosomes does not have sufficient power for our results to be considered unequivocal in proving the association between rare variants of *RTN4R* and schizophrenia [39], [60], [61]. Therefore, we cannot exclude the possibility that all four *RTN4R* non-conservative variants that have been described in patients with schizophrenia (present study and in Sinibaldi et al. [31] study) might actually be rare variants that are neutral in relation to the disease status. In general, interpreting association tests between rare variants and complex traits is an emerging problem in the field [62], [63]. The significance of rare variants such as the ones identified in this study can be addressed by screening thousands of both patient and control chromosomes for the presence of non-synonymous variants [61], by searching for rare non-synonymous variants in extended pedigrees (i.e. ones that show linkage to the 22q11.2 locus) where co-inheritance of rare non-conservative variants with disease status can be strictly evaluated, as well as by using model systems to strengthen a functional link between variation in the gene and the disease.

In the context of suggestive human genetic data, the generation of genetic mouse models that mimic the effect of rare hypomorphic variants can be particularly informative [40]. Our animal model studies, designed to facilitate interpretation of our human genetic results, indicated an effect of *Rtn4r* on locomotor activity in an open field assay. Furthermore, in heterozygous *Rtn4r* mice, we found a haploinsufficient effect in the open field assay. However, in schizophrenia-related behavioral tasks, such as PPI and the working memory-dependent T-maze delayed alternation task, our studies revealed an unremarkable behavioral profile with normal performance in these tasks, thus arguing against a major role of *RTN4R* in susceptibility to schizophrenia. It should be noted that using an independently generated *Rtn4r-*deficient mouse model, Kim et al. [64] also observed hypoactivity in *Rtn4r* null mice along with decreased rotarod performance, but normal Basso-Beattie-Bresnahan (BBB) scores, a measure of locomotor function. Schizophrenia-related behavioral tasks were not reported in the Kim et al. [64] study.

Obviously, positive results from accurate genetic mouse models can be instrumental in establishing the “biological plausibility” of disease-associated genetic variants. However, any interpretation based on negative results from behavioral studies in animal models suffers from several caveats that reflect the limitations of behavioral approaches and the complex genetic structure of the disease. For example, the behavioral effect of any given mutation, especially in a gene that could be involved in a complex disorder, could depend on the age and genetic background of the organism. In addition, we cannot ignore the possibility that *RTN4R* deficiency contributes to other phenotypic components not assayed here. In that respect, future studies designed to examine the effect of *Rtn4r* deficit in younger animals or in inbred genetic backgrounds including additional hypothesis-driven behavioral assays will be informative.

Behavioral assessment at baseline may also overlook modulatory effects of a gene in a given behavior that may be revealed by additional genetic or pharmacological challenges. NMDA hypofunction is considered one of the leading hypotheses for schizophrenia pathogenesis and has inspired the generation of several pharmacological models of the disease. Indeed, by adapting such a pharmacological model we found that neonatal NMDAR disruption during a presumably critical period for NMDA activity (P7–10), differentially affects wild-type and *Rtn4r*-deficient mice. During critical periods of development, widespread pharmacological or genetic inhibition of NMDAR signaling has been shown to facilitate terminal axon sprouting and to modulate the effects of activity-based competition on axon growth [58], [65], [66]. Such “wiring” alteration may contribute not only to the behavioral deficits associated with this pharmacological model, but also to the disease pathogenesis. Moreover, *Rtn4r* may modulate this aspect of NMDA hypofunction on early brain wiring.

Interestingly, and in agreement with our behavioral analysis at baseline, the observed effects were specific to the motor domain. Although it is very difficult to extrapolate from motor deficits in mice to deficits in motor skills in humans, it is worth noting that patients with schizophrenia display motor development delays, as well as motor disturbances [67], [68]. It is also worth noting that motor deficits have been consistently observed in children with the 22q11.2 deletion syndrome including an impairment in fine and gross motor skills that emerges during adolescence [11], [69], [70]. Importantly, a recent study has shown that motoric deficits are more prevalent among 22q11.2-deleted individuals with schizophrenia than without schizophrenia and along with deficits in verbal learning and social cognition can be used to distinguish these two groups of patients [71].

Our results do not support a major role of *RTN4R* in susceptibility to schizophrenia or in the cognitive and behavioral deficits observed in individuals with 22q11 microdeletions. However, a hypothesis emerging from the findings described here, as well as from previous independent work (see Introduction) is that *RTN4R* may modulate the genetic risk for psychiatric phenotypes in a subset of patients, at least partly by mediating the developmental effects of NMDAR receptor hypofunction on early brain wiring. Further genetic studies in afflicted families, as well as analysis of the knockout mice in additional behavioral, synaptic and molecular phenotypes are warranted and will be necessary to test this hypothesis and further clarify any role of *RTN4R* in psychiatric phenotypes.

## Materials and Methods

### Sample description

All procedures of subject recruitment and evaluation were approved by the Institutional Review Boards at Rockefeller University, New York State Psychiatric Institute and University of Pretoria. Written informed consent was obtained from all participants. The sample used for direct sequencing consisted of 208 individuals with schizophrenia, of European ancestry, recruited from the U.S. and diagnosed by a clinical team specially trained in the use of the Diagnostic Instrument for Genetic Studies (DIGS) [72] and the research application of the Diagnostic and Statistical Manual–4th Edition (DSM-IV) [73]. On the basis of the information gathered in the DIGS, the clinical interviewers assigned appropriate diagnoses according to the DSM-IV. The control sample used for direct sequencing consisted of 60 parents from CEPH trios (residents of Utah with ancestry from Northern and Western Europe) and an additional set of 240 healthy Caucasians collected from the U.S. by us (MK). Sequencing analysis was performed as described in **Text S2**.

To evaluate association between the *RTN4R* gene and susceptibility to schizophrenia, we recruited participants from 312 Afrikaner families with at least one schizophrenic subject. Afrikaners are descendants of mostly Dutch immigrants who settled in South Africa beginning in 1652 [74]. Participants were diagnosed in person by specially trained clinicians again using the DIGS, which had been translated and back-translated into Afrikaans. Patients were classified into two diagnostic categories. The stricter category, Sch1, includes 348 individuals who meet DSM-IV criteria for schizophrenia or depressed-type schizoaffective disorder. Family data suggest that the two diagnoses are alternative expressions of the same genotypes [75] and in the past we have considered them together under one phenotypic liability class, LC I [76]. The broader category, Sch2, includes an additional 52 individuals diagnosed with schizoaffective disorder of mainly affective course. Association analysis was performed as described in **Text S2**.

### Generation of *Rtn4r* knockout mice

For the construction of the targeting construct, we replaced an *Rtn4r* genomic fragment encompassing exon II with the self-excisable pACN cassette including the *neo* gene selectable marker [77]. Cell culture, embryonic stem (ES) cell electroporation and generation of the chimeric mice were performed essentially as previously described [78]. Approximately 5% of the tested ES cell clones were positive for homologous recombination, and one clone was selected for injection into C57BL/6J blastocysts. Chimeric males were mated with C57BL/6J females, and DNA from tail biopsy samples from F1 agouti-coat pups was genotyped by Southern blotting at the *Rtn4r* genomic locus (wild-type fragment: 14.6-kb; recombinant fragment after pACN cassette excision: 3.8-kb). We mated F1 heterozygous mice and obtained F2 mice of all three genotypes. The mutation was on a hybrid C57BL/6×129Sv background. All animal procedures were performed according to protocols approved by the appropriate Institutional Animal Care and Use Committee under the federal and state regulations.

### Histological analysis

See Text S2.

### Behavioral Testing: Equipment and Procedures

See Text S2.

### Analysis following transient postnatal NMDAR blockade

See Text S2.

## Supporting Information

Text S1 (0.02 MB DOC)

Text S2 (0.06 MB DOC)

Table S1 (0.03 MB DOC)

Figure S1 Brain histology in Rtn4r-deficient mice: Representative microphotographs from Nissl staining of coronal sections through the cerebral cortex (A, B), anterior-dorsal hippocampus (C, D) and corpus callosum (E, F) from 8-wk-old Rtn4r-deficient mice (−/−) and their wild-type littermates (+/+). (G, H) Coronal sections through the cerebral cortex of Rtn4r recombinant mice crossed with Thy1-YFPH expressing transgenics. Scale bars represent 0.4 mm. (I) Average cell density of the retrosplenial agranular cortex at Bregma −2.18 mm, −1.70 mm, and −1.22 mm. (J) Average thickness of the retrosplenial agranular cortex. (K) Average thickness of the corpus callosum. All data are represented as mean±S.E.M.
(9.10 MB TIF)
