# Supplementary material for: Nogo Receptor 1 (RTN4R) as a Candidate Gene for Schizophrenia: Analysis Using Human and Mouse Genetic Approaches
Source: PLoS One. 2007 Nov 28;2(11):e1234. doi: 10.1371/journal.pone.0001234 (PMC2077930; doi:10.1371/journal.pone.0001234)
Supplement: Text S1 — (0.02 MB DOC) [file pone.0001234.s001.doc]

**Text S1**

Morphometric calculations of cell densities, laminal thickness and the thickness of the corpus callosum did not reveal any anomalies (**Fig. S1: A-K**). The orientation of dendritic trees of cortical and hippocampal neurons appeared to be normal (**Fig. S1: G, H,** and data not shown). Cell density in wild-type and knockout mice was calculated at the retrosplenial agranular cortex and basolateral amygdala at Bregma -2.18 mm, -1.70 mm, and -1.22 mm (**Fig. S1: I**). Laminal thickness was measured at the retrosplenial agranular cortex and the corpus callosum at Bregma -2.18 mm, -1.70 mm, and -1.22 mm (**Fig. S1: J, K**). No differences were observed in any of these measurements.
