# Supplementary material for: Nogo Receptor 1 (RTN4R) as a Candidate Gene for Schizophrenia: Analysis Using Human and Mouse Genetic Approaches
Source: PLoS One. 2007 Nov 28;2(11):e1234. doi: 10.1371/journal.pone.0001234 (PMC2077930; doi:10.1371/journal.pone.0001234)
Supplement: Text S2 — (0.06 MB DOC) [file pone.0001234.s002.doc]

**Text S2**

**Direct sequencing:** Genomic DNA was prepared from collected blood samples using the QIAamp DNA Blood Maxi Kit (Qiagen) according to the manufacturer’s instructions. *RTN4R,* which is comprised of two exons, was sequenced in each of the 208 U.S. schizophrenia samples and 300 controls using three primers spanning exon II (5'ACACACGCTGCACCTGGAC3', 5'GTGACCTCAAACGCCTAGC3', 5'AGGAAGAGGTGTGTGAGGT3'). Exon I, which encodes for only 7 amino acids, was not examined. Sequencing was performed on an ABI 3730 using a sequencing program of 10 sec at 96 °C, 5 sec at 50 °C, 4 min at 60 °C, repeated for 25 cycles. We aligned and analyzed the sequences using Sequencher 4.5 (Gene Codes Corporation).

**Association analysis:** Genomic DNA was prepared from collected blood samples using the QIAamp DNA Blood Maxi Kit (Qiagen) according to the manufacturer’s instructions. Using the Illumina GoldenGate platform, five Single Nucleotide Polymorphisms (SNPs) in the *RTN4R* gene were genotyped to evaluate association between the *RTN4R* gene and susceptibility to schizophrenia in the Afrikaner families. To test for association with schizophrenia, we applied the transmission-disequilibrium test (TDT) to each SNP and also a parametric test of association implemented in the LAMP program (1, 2) that provides additional power when there is no population stratification. Whereas the TDT focuses on alleles transmitted from heterozygous parents to affected offspring and is thus immune to stratification, the parametric approach uses all available genotypic and phenotypic information to estimate the most likely model for association between the trait and a SNP, but it does not take stratification into account. To complement our single SNP analyses, we assigned all SNPs to a cluster and used the Expectation Maximization (E-M) algorithm implemented in Merlin (3) to estimate haplotype frequencies and to identify the most likely set of haplotypes for each family. We then tested for association between common haplotypes (frequency ≥ 0.05) and schizophrenia.

**Histology:** Eight-week-old animals were anesthetized and transcardially perfused with phosphate buffered saline (PBS) followed by 4% paraformaldehyde (PFA) in PBS. Following dissection, brains were post-fixed overnight and cryoprotected in 30% sucrose. Using a freezing microtome, 40 mm frozen coronal sections were serially collected in PBS, mounted onto Superfrost Plus slides (VWR), and stained with cresyl violet (0.1%). Images were collected using a camera mounted on a microscope (Nikon E800) equipped with Spot Software (Diagnostic Instruments, Inc). All measurements were made using ImageJ software (NIH). All analyses were performed on the corpus callosum, retrosplenial agranular cortex, and basolateral amygdala at three sections: Bregma -2.18, Bregma -1.70, and Bregma -1.22. To measure cell density, a 0.3 x 0.3 mm sampling box was used. *Rtn4r* knockout animals were also crossed with a Thy1-YFP line H mouse strain (Jackson Labs, Bar Harbor, ME), which expresses yellow fluorescent protein (YFP) in a low percentage of a variety of neurons, including cortical layer V neurons and hippocampal pyramidal neurons. Eight-week-old animals were anesthetized and transcardially perfused with PBS followed by 4% PFA in PBS. Following dissection and overnight fixation, 75 mm coronal sections were cut using a microtome and serially collected in PBS. Sections were mounted onto Superfrost Plus slides (VWR) and preserved in Vectashield anti-fading reagent (Vector Laboratories) before visualization using a fluorescence microscope (Nikon E800).

**Behavioral Testing: Equipment**

*Open field, Light/Dark, Fear conditioning*: For the open field, light/dark, and fear conditioning tests, the Hamilton-Kinder SmartFrame^TM^ system was used (Hamilton-Kinder, Poway, CA). The basic system consisted of a standard 4 x 8 photobeam array system and a standard 4 x 8 photobeam rearing frame. Different inserts were used to accommodate the open field, light/dark and fear conditioning tests. The Plexiglas open field insert measured 24.13 cm (W) x 18.0 cm (L). The light/dark insert was of the same overall dimensions except that one half of the box was clear Plexiglas and the other half was black Plexiglas. The two halves were separated by a manual guillotine door which, when removed, allowed the animal to move freely between the two compartments. The fear conditioning insert measured 24.13 cm (W) x 9.0 cm (L) and had a grid floor connected to a shock generator. The top of the box included a speaker attached to a sound generator.

*Analgesia:* The Hotplate Analgesia meter (Model 39) was manufactured by IITC Inc. The unit had an anodized plate measuring 11" x 10.5" x 0.75”. A heat sink compound (Radio Shack® Cat. no. 273-1372) was used between the hotplate surface and aluminum plate to facilitate an even distribution of heat. The aluminum plate was held in position with binder clips. A small, bottomless smoke grey Plexiglas enclosure was used to confine animals to the center of the aluminum plate.

*Acoustic Startle/Prepulse Inhibition:* A HamiltonKinder SM100 Startle Monitor was used. The system consisted of a base plate, mouse sensing plate, mouse restrainer, and Newton impulse Calibrator (calibrated in newtons) enclosed in a sound attenuating cabinet. The cabinet measured 14” x 10.875” x 19.5” and had 35 decibels of attenuation. The system was designed to provide + 1 dB accuracy on a scale from 57–120 dB. The system was also designed to minimize variability between test chambers (< 1 dB) ([www.hamiltonkinder.com](http://www.hamiltonkinder.com/)).

*Tail suspension*: The tail suspension apparatus was manufactured by MedAssociates, St. Albans, VT. Each unit consisted of a linear load cell, load cell amplifier, and filter, which were connected to a transducer to which the animal's tail was taped. Each unit was enclosed in a cubicle.

**Behavioral Testing: Procedures**

**Analysis at baseline:** Twenty mice of each genotype (10 males and 10 females) underwent a battery of tests in the following order: Day 1: Open field, Hotplate; Day 2: Light/Dark, Startle, Fear Conditioning: Training Day; Day 3: Contextual Conditioning, Cued Conditioning, Tail suspension. An independent sample of equal size (10 males and 10 females of each genotype) was used for the T-maze delayed alternation task. On days when multiple tests were performed, tests were separated by approximately 2–2.5 hrs, including a habituation period prior to each test. To avoid stressing animals on testing days we painted the tails of the mice using Sharpie® permanent markers to allow easy identification of the appropriate animals with minimal disturbance to the cage mates. All animals were weighed (to the nearest 0.1 g) using a scientific balance.

*Open Field:* Animals were placed in the center of the open field to begin the test. During the 20-minute session (divided into four-5 min intervals), infrared beams detect horizontal movements and vertical beams detect vertical movements; this information is transmitted to an analyzer. Total distance traveled, rears, intra-session habituation, and percentage of time spent in the center of the open field during the test session were evaluated. Intra-session habituation was calculated by taking the ratio of activity during the last 5-minute interval to distance traveled during the first five plus last five minutes of the test session.

*Hotplate:*  A standard hotplate testing protocol was used to assay nociception. All overhead lights were turned off 1 hr prior to hotplate testing. A small desk lamp with the light (15 W bulb) directed away from the hotplate was used so that the tester could observe the animal. A mirror was also located directly behind the hotplate so that if the mouse was facing away from the tester, the tester could still observe pain responses. The hotplate was preheated to 52 °C. The temperature was verified using a surface thermometer. The mouse was placed in the center of the hotplate within the confines of the Plexiglas enclosure. We observed pain responses primarily associated with the hind paws including licking, shaking, guarding, and lifting. Latency to respond was measured; if the animal did not show a pain response within 20 sec, the test was discontinued.

*Light/Dark:* A lamp with a 15 W bulb was located directly above the light portion of the light/dark box. To begin the 10 min test, animals were placed in the light half of the box. The guillotine door was then removed to allow animals to freely move between the two halves of the box. The amount of time spent in the light versus dark compartment was measured.

*Acoustic Startle and Prepulse Inhibition:* Animals were placed in the startle chamber with a 65 dB background white noise and allowed to habituate. Over an approximately 15 min session, 55 pseudo-random trials were given. A 120 dB white noise burst was used as the acoustic startle stimulus. Pre-pulses were 70, 80 and 85 dB white noise bursts, which preceded the startle stimulus by 10 ms. Startle response to the startle stimulus and to each of the pre-pulse dB levels was measured. Pre-pulse inhibition was measured using the following formula: [100- (pre-pulse startle/acoustic startle) x 100].

*Fear Conditioning:* Training: Animals were placed in the fear conditioning chamber and allowed to habituate for 2.5 min. Animals were then presented with three pairings of an 85 dB tone and 0.36 mA foot shock. The tone was 30 sec in duration and the shock was presented during the last 2 sec of the tone. There was a 2.5 min interval between each of the tone + shock pairings. Because this test was automated, beam breaks were measured in 30 sec intervals. Contextual Conditioning: On the day following the training session, animals were placed back into the same chambers where they underwent training. During this 6 min session, activity (beam breaks) per 30 sec bin was measured and compared to activity during the habituation period on the training day. This procedure was used as a measure of conditioning to the context. Cued Conditioning: Approximately 2 hrs later, the behavior of the mice was tested in an altered context. The fear conditioning chambers were altered by placing a gray, square tile over the grid floor, placing a black Plexiglas insert over the walls of the chambers, and attaching a small cup containing orange oil diluted in water in the upper corner of the box. Animals were allowed to explore the altered environment for 2.5 min, after which time the conditioned stimulus (tone) was presented for 2.5 min. Activity (beam breaks) was evaluated in 30 sec bins. Activity suppression during presentation of the tone was evaluated relative to the activity during the habituation period in the altered context.

*Tail Suspension:* Mice were suspended by the tail with tape attaching them to the transducer. During the 6 min test, force of movement, or lack thereof, was recorded and reflected the time the animal spent immobile during the test.

*T-maze delayed alternation task:* A discrete paired-trial delayed alternation version of the traditional T-maze task was used to assay working memory. Briefly, mice were kept on a maintenance diet throughout the course of all T-maze experiments at 85-90% of their normal body weight. Animals were given three adaptation sessions (one per day) during which they were allowed to freely explore the T-maze and obtain food rewards. Shaping sessions were administered the following two days: mice were subjected to one free choice trial (both T-maze arms were baited) and 10 forced alternation trials (5 left and 5 right) in which access to the previously visited arm was blocked. Mice were confined to the starting compartment for 5 sec between trials. After 5 days of adaptation and shaping, mice were subjected to the test sessions (one per day). Test sessions consisted of 11 free choice trials with rewards given for alternating arm choices; arm choices were recorded. Upon reaching 80% accuracy (80% alternating arm choices), each mouse was administered a test session in which the delay between trials was increased from 5 to 20 sec. Mice were returned to their home cages after their daily training.

**Analysis following transient postnatal NMDAR blockade:** All pups received subcutaneous injections twice a day for four consecutive days of either MK-801 (0.25 mg/kg) or saline. Pups were injected at either postnatal day 7–10 (P7–10) or P11–14, and each injection group consisted of 8 to 10 animals of mixed sex. Pups were weighed daily during the injection period and weaned at P22.

*Spontaneous alternation:* At P62 and P63, all animals were tested for spontaneous alternation using a Y-maze procedure. Animals were placed in a symmetrical Y maze arena and allowed to roam freely for eight minutes; each animal was tested for two consecutive days. The sequence of arm entries was recorded and movement was tracked using a videotracking system (Ethovision 3.0). The percentage alternation was calculated as [number of alternations/(total arms entered -1)].

*Acoustic startle and prepulse inhibition:* At P91, animals were tested for startle response and PPI. Animals were placed in the startle chamber with a 65 dB background white noise and allowed to habituate. Over an approximately 15 min session, 55 pseudo-random trials were given. A 40 ms, 120 dB white noise burst was used as the acoustic startle stimulus. Prepulse stimuli were 65, 72, and 77 dB white noise bursts, which preceded the startle stimulus by 100 ms. Response to the startle stimulus and to each of the prepulse dB levels was measured. Percentage PPI was measured using the following formula: [100 - (prepulse startle/acoustic startle) x 100].

**References:**

1. Li M, Boehnke M, Abecasis GR (2005). Joint modeling of linkage and association: identifying SNPs responsible for a linkage signal. *Am J Hum Genet*. 76:934–949.

2. Li M, Boehnke M, Abecasis GR (2006). Efficient study designs for test of genetic association using sibship data and unrelated cases and controls. *Am J Hum Genet* 78:778–792.

3. Abecasis GR, Wigginton JE (2005). Handling marker-marker linkage disequilibrium: pedigree analysis with clustered markers. *Am J Hum Genet* 77:754-767.
