# Supplementary material for: Nogo Receptor 1 (RTN4R) as a Candidate Gene for Schizophrenia: Analysis Using Human and Mouse Genetic Approaches
Source: PLoS One. 2007 Nov 28;2(11):e1234. doi: 10.1371/journal.pone.0001234 (PMC2077930; doi:10.1371/journal.pone.0001234)
Supplement: Table S1 — (0.03 MB DOC) [file pone.0001234.s003.doc]

**Table S1: Study population characteristics**

**(genotyped individuals only)**

|  | **Sch1** | **Sch2** |
| --- | --- | --- |
| Affected individuals  Unaffected individuals | 348 (29%)  852 (70%) | 400 (33%)  800 (66%) |
| Affected females  Affected males | 124 (36%)  224 (64%) | 146 (37%)  254 (63%) |
| Families with:  ≥1 affected individual  ≥2 affected individuals  ≥3 affected individuals | 282 (68%)  51 (12%)  12 (3%) | 312 (76%)  67 (16%)  17 (4%) |
| Average affected individuals per  family with ≥2 affected individuals | 2.38 | 2.36 |
