# Supplementary figures and images for: Nogo Receptor 1 (RTN4R) as a Candidate Gene for Schizophrenia: Analysis Using Human and Mouse Genetic Approaches
Source: PLoS One. 2007 Nov 28;2(11):e1234. doi: 10.1371/journal.pone.0001234 (PMC2077930; doi:10.1371/journal.pone.0001234)

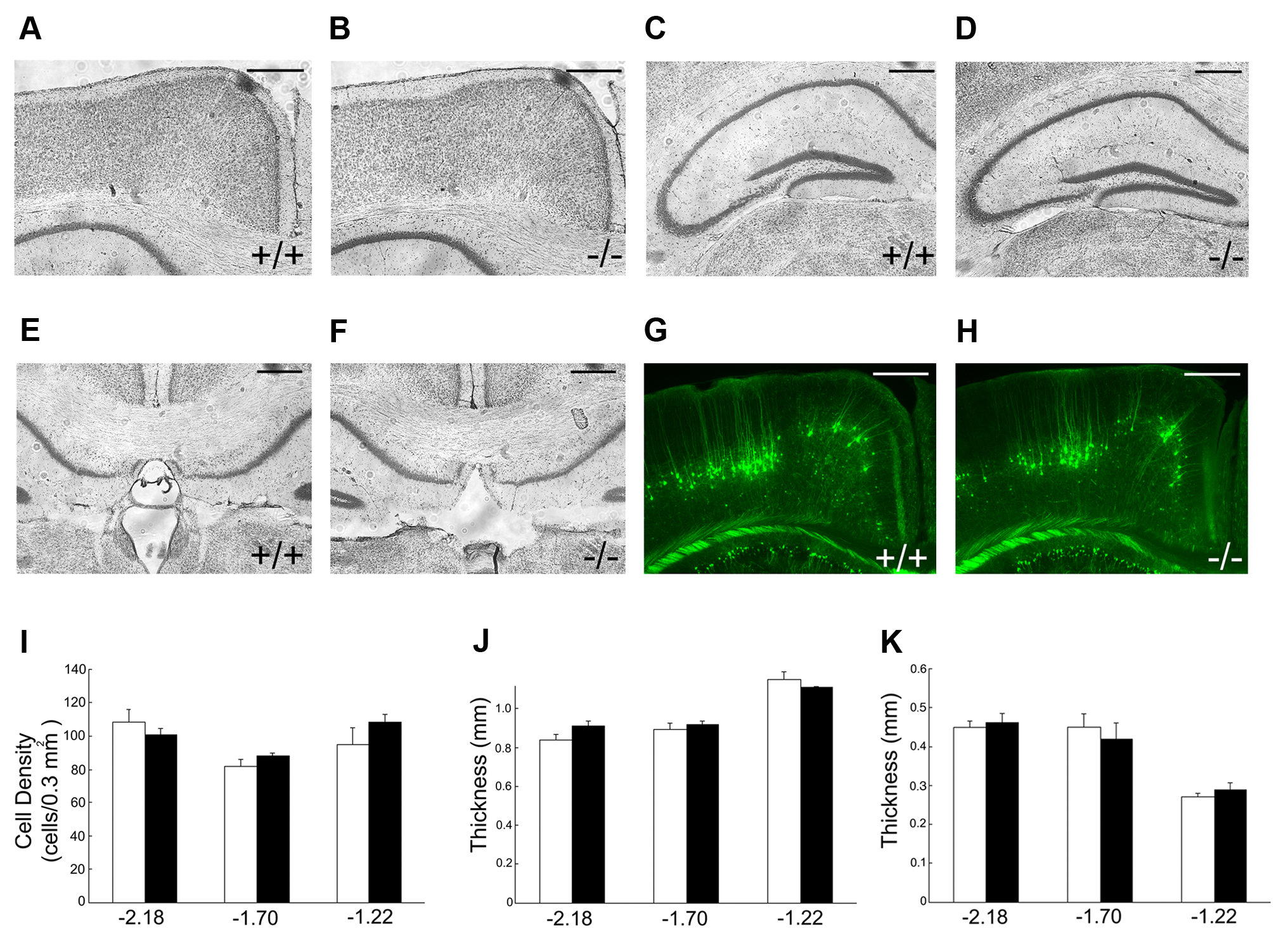

Supplement: Figure S1 — Brain histology in Rtn4r-deficient mice: Representative microphotographs from Nissl staining of coronal sections through the cerebral cortex (A, B), anterior-dorsal hippocampus (C, D) and corpus callosum (E, F) from 8-wk-old Rtn4r-deficient mice (−/−) and their wild-type littermates (+/+). (G, H) Coronal sections through the cerebral cortex of Rtn4r recombinant mice crossed with Thy1-YFPH expressing transgenics. Scale bars represent 0.4 mm. (I) Average cell density of the retrosplenial agranular cortex at Bregma −2.18 mm, −1.70 mm, and −1.22 mm. (J) Average thickness of the retrosplenial agranular cortex. (K) Average thickness of the corpus callosum. All data are represented as mean±S.E.M. (9.10 MB TIF) [file pone.0001234.s004.tif]
